# Examining the Effects of Overtime Work on Subjective Social Status and Social Inclusion in the Chinese Context

**DOI:** 10.3390/ijerph17093265

**Published:** 2020-05-07

**Authors:** Yashuo Chen, Pengbo Li, Chunjiang Yang

**Affiliations:** 1 College of Business, Yantai Nanshan University, Yantai 265713, China; chenyashuo@stumail.ysu.edu.cn; 2 School of Economics and Management, Yanshan University, Qinhuangdao 066004, China; 3 School of Tourism Sciences, Beijing International Studies University, Beijing 100024, China

**Keywords:** overtime work, subjective social status, social inclusion, fairness, overtime type

## Abstract

Although researchers have argued that long work hours have been shown to threaten individual health, lead to work-family conflict, and reduce job performance, the effect of overtime work on social-related outcomes has received little attention. Based on the framework of relative deprivation, we attempt to address this important issue by exploring whether, why, and when individuals’ overtime work influences their social attitudes. By using the data of 400 Chinese employees from the China Labor-Force Dynamics Survey (CLD), we found that overtime work was associated with a low level of subjective social status and social inclusion. In addition, we found that the time type of overtime work (work overtime on weekdays or on weekends and holidays) has a moderating effect on the relationship between overtime work and social inclusion. That is, employees who work overtime on weekdays are unlikely to have a sense of social inclusion. Furthermore, the negative relationship between overtime work and subjective social status was stronger at a low level of fairness rather than a high level of fairness. In contrast, the negative relationship between overtime work and social inclusion was stronger at a high level of fairness rather than a low level of fairness. These findings highlight the critical role of overtime work in social life and also provide novel insights into social intervention aimed at the happiness and harmony of a society.

*“They won’t let us go unless we have everything finished. So we have to work overtime···. If we didn’t finish the work even in 10 h, we stay until 2 a.m. We have to finish the work.”*

Statement of a food packer worker [1]

## 1. Introduction

In 1940, the United States’ Fair Labor Standards Act established the 40-h workweek and laid a foundation for the idea that a traditional work schedule would roughly run eight hours, from 9 a.m. to 5 p.m., Monday through Friday. However, the reality is that many employees around the world have work schedules that do not conform to such parameters. Indeed, organizations increasingly need employees to work overtime during the day and week. Nowadays, overtime work has become a common phenomenon all over the world. For example, Parent-Thirion et al. showed that 15% of workers in the European Union (EU) countries habitually work 48 h or more per week, and in Turkey, 56% of workers work more than 48 h/week [2]. According to the Current Population Survey (CPS) data, one-quarter of Americans work more than 40 h per week [3,4]. Asia, in particular, seems to have the worst overtime situation. In China, for example, the work time of people in urban areas is up to 46.2 h a week [5]. Although these international figures provide a rough picture of the prevalence of overtime work worldwide, they underestimate all overtime work being performed, as the figures focus on the prevalence of overtime work (in many reports defined as over 40 h/week) instead of reporting the accuraty of overtime hours [6]. According to a survey of migrant workers in China, the average working hours per week of the respondents were 63.1 h (Standard Deviation, SD = 17.8) [7]. 

Importantly, employees have long been plagued by overtime work. A common finding of the growing number of empirical studies conducted in different societal contexts is that overtime work is associated with some significant health-related and personal/family-related outcomes. The considerable body of existing research has shown that long work hours increase the risks of cardiovascular diseases [8,9,10], chronic fatigue, stress [11], depressive state, anxiety, insomnia, all-cause mortality, alcohol use, and smoking [8], and also influence self-perceived health, mental health status, hypertension, and other health behaviors [12]. Similar findings have been found by other researchers, for instance, overtime work leads to myocardial infarction, poor physical health and injuries, alcohol consumption, smoking, physical inactivity, and depression [11]. Long-term work overtime employees also make reconciling work with other parts of life more difficult. In the sixth European working conditions survey, subjects who worked 48 h or more per week were more likely to experience work–life conflict and health problems compared with those who worked standard hours. Workers who report long working hours are four times less likely to maintain a good balance between working hours and social commitment. They are more likely to perceive health and safety risks and feel exhausted at the end of the workday [2].

In spite of the volume and expanse of the research noted above, there remains much to be clarified regarding overtime. First, previous studies on overtime work always highlighted the adverse effects of overtime work on an employee’s health and family. However, general literature has not paid sufficient attention to the social implications of overtime work [13]. In fact, work hours have always been the core of people’s social life, and a certain amount of work hours was not only a necessary prerequisite for ensuring household income, but also an important factor infusing the perception of social status. In addition, ignoring the mechanism of work hours and its scope of influence made it difficult to understand the nature of work hours, systematically and comprehensively [14]. To the best of our knowledge, the relationship between work hours and social status and social inclusion has not yet been explored. Therefore, in this study, we try to understand how overtime affects an individual’s evaluation of social status and social inclusion. This may help to open a new door for future overtime research.

Second, most recent studies on work overtime concentrated on developed countries [14], such as the United States [15] and Japan [16], resulting in insufficient awareness of the effects upon developing countries. The fact is that many findings have indicated that the overtime work in developing countries is more widespread, and employees suffer from more time-related stress [17], which lead to more serious psychological and sociological implications. With the rapid growth of the economy, China has become the second-largest economy in the world. In China, with the improvement of globalization and the rapid development of the service industry, most employees, including blue-collar workers, administrators, managers, and professionals, have long working hours and high work intensity. Long hours of work disturb people’s pace of life, which is not conducive to personal perception of social identity and social inclusion. However, there are few studies on overtime in the Chinese context. Although excessive labor and its related consequences (such as social interaction, social status, social inclusion, and exclusion) have attracted great attention by the media and managers [18], with the exception of sweatshop employers, little is known about the problem of employees working long hours in China. Thus, it is important to give close attention to overtime work in the Chinese context.

Third, most studies basically investigated the simple, direct association between overtime and health-related or family-related outcomes. Thus, overtime was treated as a “black box”. Perhaps unsurprisingly, this focus on gross correlations did not lead to a clear description of the relationship between overtime work and employee outcomes. A better way to understand the complexity of overtime is to study the impact of potential mediators. For example, several studies in the Netherlands have shown that the link between overtime and health does not seem to be simple and direct, but rather depends on the profile of working hours and the psychosocial characteristics of workers [19]. Almost all of the existing studies have provided only general figures concerning the prevalence of overtime. Although scholars recognize the importance of "time background" [20], little is known as to exactly how overtime works [21]. Timing norms, which people experience as socially shared, expected patterns of paced activity, governs various activities in organizations and life [22]. Generally speaking, given that weekends and holidays represent a particular time for ‘‘fun, pleasure, and entertainment,’’ work on weekends and holidays destroys individuals’ participation in timing norms. We, therefore, aim to fill this void by examining the effects of overtime type in the relationship between overtime and subjective social status and social inclusion. A more fine-grained analysis of overtime in its natural context to develop a more-detailed work picture of holidays may provide insights into some fundamental issues on overtime. In addition, people’s cognition, attitude, and behavior in social interaction are closely related to their views on justice or injustice. Without considering the importance of the fairness of the social environment, we may not be able to fully explain the effects of overtime on peoples’ social attitudes and behaviors. Accordingly, we contribute to the work time literature by investigating the moderating effects of social fairness on the relationship between overtime work and subjective social status and social inclusion. 

## 2. Theoretical Framework and Hypotheses Development

### 2.1. Theoretical Framework

This study is based on the theoretical framework of relative deprivation theory. Relative deprivation (RD), as a social evaluation theory, is defined as a judgment that one or one’s ingroup is disadvantaged compared to a relevant referent and that this judgment invokes feelings of anger, resentment, and entitlement [23]. According to Smith and his colleagues’ research [24], RD includes four fundamental components: (1) people often engage in cognitive comparisons, (2) these cognitive appraisals lead people to perceive that they or their inner groups are disadvantaged, (3) people recognize that these disadvantages are unfair, (4) people resent these unfair and undeserved disadvantages. The intuitive explanatory appeal of RD has led to its use across numerous social science disciplines. Researchers have invoked this concept to explain social phenomena ranging from well-being to participation in collective protests [24].

RD assumes that when individuals’ expectations of the goods and living conditions to which they feel they are entitled are not met, they become angry and motivated to correct what people think is unfair [25]. Unfair perceptions and resentments arise from social comparisons (interpersonal and intergroup) made by individuals. Generally speaking, an individual’s relative deprivation includes two aspects: cognition and emotion [26]. Cognition refers to the recognition or perception of disadvantages [27]. It relates to the extent to which people recognize that they have been disadvantaged by a situation of which they disapprove. The affected ones focus on the degree to which people express resentment and anger at situations that they believe deprives them relative to other members [28,29]. These two kinds of relative deprivation are the result of the comparison between the disadvantaged groups and the dominant groups in terms of their situation, status, outcomes and privileges. People who perceive unjust and hateful disadvantages may respond in a variety of ways, including by opposing deprivation and avoiding stimulating environments [26].

### 2.2. Overtime Work and Subjective Social Status

The relative deprivation theory explains the individual-level mechanisms underlying the relationship between overtime work and individuals’ perceived social status. This theory suggests that a disadvantage manifests itself through various forms of socio-economic comparisons, particularly the inequality between inputs and outputs. These different forms of socioeconomic comparisons, in turn, make individuals think about their social identity and well-being, and more generally, ultimately lead to social exclusion [30]. It therefore seems that the employees who regularly engage in overtime work are more likely to perceive relative deprivation and then may engage in underestimating self-categorization behaviors that have a prejudicial effect on their social status [31].

Specifically, people prefer to get positive self-evaluation based on their own comparison with others. They try to differentiate themselves positively from others. An important source of self-evaluation is the relative position of the group people belong to and identify with. Based on the results of comparison with relevant external groups, such status may be characterized as favorable or unfavorable. Furthermore, a disadvantaged or inferior position in one’s group will lead to a “negative social identity.” An individual’s social identity includes a cognitive component and an evaluative component. The former refers to beliefs about one’s self that can pertain to one’s social status; the latter concerns one’s value. Social identity collectivizes the individual by categorizing the individual self as an inclusive social class and stimulates the sharing of identity between the individual and the group [32]. In other words, overtime employees can put themselves and other overtime workers into the same social class. To assess their personal and group status, individuals compare themselves or their group with others. The motivation to feel positive about one’s social group and one’s self renders individuals sensitive to unfavorable comparisons. It is through those arguments that the relationship between RD and social identity has been posited and tested [33]. Compared with other social groups who do not work overtime, those who work long hours would be at a disadvantage, feel relative deprivation, and thus identify with lower social status. In addition, existing research on RD, social identity, and social comparison shows that people with lower relative deprivation will have a negative evaluation of their social identity. Therefore, we hypothesized the following:

**Hypothesis** **1:** Overtime work will be negatively related to individuals’ subjective social status.

### 2.3. Overtime and Social Inclusion

To analyze the relationship between overtime and social inclusion, we can start from the objective and subjective perspectives. From the objective point of view, due to limited time, long work hours make employees unable to carry out necessary social activities. Evidence from China shows that people can spend a lot of time in interpersonal communication to maintain their social role. Specifically, Banerjee and Duflo suggested that high time investment of Chinese people in holidays and festivals serves as an essential social function, and the consequences of refusing to participate are grave [34]. There is a proverb that says you can’t have your cake and eat it too. People who work too long spend less time on social activities, which affects the social inclusion of individuals in the community. Most approaches to social exclusion place particular emphasis on the relationship between employment and social capital [35]. From this perspective, long-term work is believed to entail sparse social networks, and it often leads to discouragement and exclusion from social activities [35].

From the subjective point of view, as mentioned above, employees who work overtime may produce the perception of relative deprivation in the social comparison process. Individuals evaluate their social identity by comparing member groups and other reference groups. If the assessment is negative, people who work long hours experience relative deprivation and are motivated to have attitudinal and behavioral responses, including separation from the original group or feeling exclusion by other groups. The theory of the need to belong argues that people have to be included in meaningful social relationships to satisfy their need to belong [36]. One of the most obvious threats to meeting the need for a sense of belonging is social exclusion. Some studies have shown that social inclusion and exclusion are related to fair or unfair behavior. It has been argued that reasonable work arrangements may convey symbolic messages of inclusion in a group, whereas unfair treatment may convey symbolic messages of exclusion from a group [37]. Thus, the relative deprivation may affirm an individual’s level of inclusion in social groups. Reasonable work arrangements can help form positive and stable intragroup relationships, while long hours of work can easily damage the disintegration of groups or associations. Under the same logic, reasonable work time arrangement can maintain the function of individuals’ integration into relevant social groups and avoid the negative consequences related to social exclusion. Therefore, we hypothesized the following:

**Hypothesis** **2:** Overtime work will be negatively related to individuals’ social inclusion.

### 2.4. Time Type of Overtime as a Moderator

In China, the overtime culture has always been prevalent. In particular, "996" (9 a.m.–9 p.m., six days a week) has even become the standard work schedule for most high-tech enterprises in Beijing, Shanghai, Guangzhou, and Shenzhen. On April 11, 2019, Jack Yun MA (founder and director of Alibaba Group), who focuses on overtime culture, said in an exchange speech with Alibaba’s internal employees: “it’s great to work in ‘996’.”. According to our classification of overtime hours, in the “996” work schedule, we work 72 h a week, of which 4 * 5 h are overtime on weekdays and 12 * 1 h are overtime on weekends and holidays.

Influenced by the “work hard” and “busyness” culture, many employees do not complain about working hours. They are not only under pressure in all aspects of their work, but also silently endure the increasing overtime and long commute time [38]. These workers spend an amazing amount of time at their jobs. It is not strange for a workaholic to work at home or to go to work on weekends and holidays. However, as far as the majority of Chinese employees is concerned, they are under high stress, and their feeling of loss of authority is considerably high because of their long working hours and mandatory work on holidays. Family activities can play a salutary role in terms of enhancing relationships and improving patterns of communication among family members [39,40]. Holman and other scholars have documented that family time and couple time are associated with family satisfaction, cohesion, adaptability, and stability [40,41]. It is evident from some literature that many couples take their responsibility for family leisure very seriously [42,43]. Shaw and Dawson disclosed that both husband and wife agree that family time and family leisure are essential [44]. It is critically important for children to spend time with their parents and do activities together, such as playing games, going for walks or bike rides, or eating out with the family [43]. Couples have to spend a lot of time in paid work activities, resulting in less time with their families after work. On the one hand, they seek ways to fulfill their work responsibilities. On the other hand, they need time to engage in free-time activities with their family and community.

Nowadays, because of the high level of time stress within many families, after-working hours have been a particularly significant time of family leisure [43]. Spare time provides time away from many routine responsibilities for working parents, including time away from the workplace and housework [45]. In addition, parents often make it a commitment to their children or their families to spend holidays with their families. They believe that parents have a responsibility to find a way to have a good time with their families, no matter how busy their lives [43]. As a result, working overtime can make employees’ family needs unmet, increasing their sense of relative deprivation. Based on the views of Bellani and D’ Ambrosio [46], there is a close relationship between relative deprivation and social exclusion. Social exclusion depends on the extent to which an individual can associate and identify with others. If a person’s deprivation persists or worsens over time, he may feel socially excluded. Chinese culture is different from western culture in terms of vacation and leisure. For Chinese families, shared time with family members is concentrated after work during the weekdays, rather than during the weekends and holidays. Therefore, if work occupies the family’s leisure time, the negative impact of overtime on the social inclusion of the subjects would be more prominent.

On the other hand, economic theory suggests that beyond a certain wage level, more income will cause workers to work less [47]. Research on happiness shows that earning a higher income can make people work less and have more leisure time [48]. Moreover, lottery winners work less and spend more time on holidays after receiving their prize [49], and the upper class spend most of their yearly expenditures on holidays and leisure travels [47]. Social stratification studies regard the function of leisure as a status symbol [50]. That is, vacation time is a label of one’s position on the “real” stratification ladder. Best found that workers prefer to take additional free time in the form of more extended weekends or vacations, for example, an extension of already available free time [51]. Thus, compared with working overtime on weekdays, it is easier for employees to perceive more significant relative deprivation on vacation. In addition, the stronger the sense of relative deprivation, the easier it is for employees to put themselves into a lower social status. Therefore, we hypothesized the following:

**Hypothesis** **3:** The time type of overtime will moderate the negative effects of overtime work on subjective social status. Specifically, when working overtime on weekends and holidays, the negative effect of working hours on subjective social status will be stronger rather than that of working overtime on weekdays.

**Hypothesis** **4:** The time type of overtime will moderate the negative effects of overtime work on social inclusion. Specifically, when working overtime on weekdays, the negative effect of working hours on subjective social status will be stronger than that of working overtime on weekends and holidays.

### 2.5. Perceptions of Effort-Reward Fairness as a Moderator

Social justice is an important issue to understand social behavior and explain how perceived exploitation affects people’s response because perceived justice and injustice have a significant impact on people’s behavior [36]. When employees often work overtime, they will evaluate the rationality of the work arrangement and argue that society be held accountable for the predicament. In particular, employees’ perceptions of global equity will be a heuristic device for explaining the specific treatment and for deciding how to respond. We expect RD theory to help us understand the impact of a sense of fairness on employee response. Suppose an employee receives an overtime arrangement. This employee may not only be concerned about the fairness of the work arrangement but also invoke causal attribution to explain who or what is responsible for the arrangement. Previous research emphasized that employees’ previous views on overall social equity can serve as a heuristic tool in evaluating the situation [52]. Imagine if an employee thinks that society is generally fair, and he/she tends to attribute overtime to the social environment, so he/she thinks that hard work is the responsibility of every member of society. However, if the organization or manager is considered responsible, the employee attributes overtime to his/her (or his/her group’s) special treatment at work. This attribution result will increase the subject’s perception of relative deprivation and, in turn, low social status. In sum, when employees think society is fair, they are not easily affected by overtime work. In contrast, when employees have established the perception that society is unfair, they will be highly sensitive to work overtime. Therefore, we hypothesized the following:

**Hypothesis** **5:** Effort-reward fairness will moderate the negative relationship between overtime work and subjective social status. Specifically, the relationship will be more salient when fairness perceptions are low rather than high.

In addition, fairness judgments become more influential when people face uncertainty [53]. According to Lind and Van den Bos’s statement, fairness is more useful in adverse conditions because fairness provides people a way to cope with dilemmas. A closely related implication is that if people are treated fairly in those difficult situations, the potential negative psychological consequences associated with overtime may be reduced. In the context of overwork, on the one hand, information about effort-reward fairness provides an essential guide for employees to direct their attitudes and behaviors that are needed to deal with such workaholic situations. We expect that when employees think there is unfair work in the organization, extra working hours will lead to more negative reactions (for example, reducing social inclusion). On the other hand, fairness can regulate people’s emotional responses to a stressful work environment. Workaholic situations can trigger the stress and negative emotions of victims. We expect that individuals who have fairness perception are able to maintain positive effects and motivation in workaholic situations. Therefore effort-reward fairness (unfairness) can attenuate (strengthen) the association of extra working hours with negative emotional and cognitive reactions. We therefore propose the following hypothesis:

**Hypothesis** **6:** Effort-reward fairness will moderate the negative relationship between working overtime hours and social inclusion. Specifically, the negative relationship between work hours and social inclusion will be stronger at a low level of fairness rather than at a high level of fairness.

**Figure 1:**
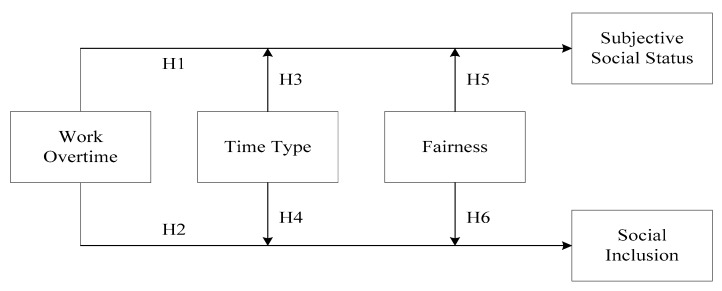
Hypothetical model.

Overall, Figure 1 illustrates our conceptual model.

## 3. Method

### 3.1. Data Source and Description

The data used in this paper were obtained from the China Labor-Force Dynamics Survey, which is organized and implemented by the social science investigation center of Sun Yatsen University. The purpose of this project is to systematically monitor the changes in the social structure, family, and labor force by tracking and collecting the information of the village, family, and individual in China. The project conducts a dynamic follow-up survey every two years in 29 provinces and regions of China (excluding Hainan, Tibet, Hong Kong, Macao, and Taiwan). It develops three main types of questionnaires, namely, village residence, family, and individual labor force, and covers various topics such as education, work, migration, health, social participation, and infrastructure. This paper uses the data of the individual labor force survey in 2016.

Based on the variables used in the study, we deleted the samples with missing values of those variables used in our study and finally, 400 valid samples were obtained. The average age was 35.62 years; 36% were aged 30 or below, 34.3% were aged between 31 and 40, and 29.7% were aged 41 and above. Among the samples, 61.3% were male, and 25.3% were unmarried. In terms of education, high school education or below accounted for 60%, junior college education accounted for 21.3%, undergraduate education accounted for 16.3%, and graduate education accounted for 2.5%. The average annual income was 57,390 Yuan. 

### 3.2. Measures

1. Overtime work. Overtime work was measured using the overtime working hours in the past month.
2. Overtime type. We divided the type of overtime into two types: overtime work on weekdays and overtime work on weekends and holidays.
3. Fairness. The questionnaire measures the effort-reward fairness by comparing the current living standard of the respondents with their work efforts. A five-point scale was adopted to measure the effort-reward fairness, ranging from 1 (totally unfair) to 5 (totally fair). The higher the score, the greater the effort-reward fairness.
4. Subjective social status. In the questionnaire, the subjective social status was directly measured by the social status rating of the respondents. Social status was graded by 10 points. From 1 (the lowest level) to 10 (the highest level), the higher the score, the higher the subjective social status.
5. Social inclusion. Social inclusion was measured by the willingness to settle locally in the future. A five-point scale was adopted, ranging from 1 (very unlikely) to 5 (very likely). The higher the score, the more willing to settle in the future.
6. Control variables. Participants’ gender, age (in years), marriage, annual income, residence type, and overtime location were treated as control variables in the study. A description of these variables is shown in Table 1.

### 3.3. Data Analysis Method and Model Estimation

To test our hypotheses, we specify and estimate the following general form regression models. We start with the specification and testing of the possibility of negative linearity (H1) in the relationship between overtime (Oi) and employee subjective social status (Si), as follows:

Si = β0 + β1 Oi + µei (1)

Similarly, we set up Equation (2) to test the possibility of negative linearity (H2) in the relationship between overtime (Oi) and employee social inclusion (ii), as follows:

ii = β0 + β1 Oi + µei (2)

Next, we test hypotheses 3 and 5 for the moderating roles of time type (Ti) and fairness (Fi) in influencing the Oi-Si relationship. We follow the approach of Baron and Kenny (1986) for testing the moderator roles of time type and fairness by adding the moderator variables (Ti) and (Fi), respectively, to Equation (1) as follows:

Si = β0 + β1 Oi + β2 Fi + β3 (Oi × Ti) + µei(3)

Si = β0 + β1 Oi + β2 Fi + β3 (Oi × Fi) + µei(4)

We also test hypotheses 4 and 6 for the moderating roles of time type (Ti) and fairness (Fi) in influencing the Oi-ii relationship, respectively, as follows:

ii = β0 + β1 Oi + β2 Fi + β3 (Oi × Ti) + µei(5)

ii = β0 + β1 Oi + β2 Fi + β3 (Oi × Fi) + µei(6)

where β is regression coefficients to be estimated, and β3 is the coefficient of the interaction terms. The moderation hypotheses (H3), (H4), (H5), and (H6) are supported if β3 is statistically significant. Equation (1) tests the direct and linear association between overtime and subjective social status. Equation (2) tests the direct and linear association between overtime and social inclusion. Equation (3) tests the moderating role of time type on the overtime-subjective social status relationship (H3). Equation (4) tests the moderating role of fairness in the overtime-subjective social status relationship (H5). Equation (5) tests the moderating role of time type on the overtime-social inclusion relationship (H4). Equation (6) tests the moderating role of fairness in the overtime-social inclusion relationship (H6). In estimating the full models represented by Equations (1) and (2), we also include appropriate control variables and use hierarchical regression analyses in SPSS 21(from IBM, Armonk, NY, USA). In addition to testing the significance of regression coefficients, we interpreted the changes in R^2^ associated with each step. We use Mplus version 7.4 [54] to examine the moderating roles of time type and fairness in the relationship between overtime and subjective social status and social inclusion. To avoid multicollinearity and to increase interpretability, we grand-mean-centered overtime and fairness prior to computing interaction terms [55].

## 4. Results

The means, standard deviations, and bivariate correlations among variables in the study are shown in Table 2. Overtime was negatively correlated with subjective social status (*r* = −132, *p* < 0.01). Overtime was negatively correlated with social inclusion (*r* = −0.204, *p* < 0.01). These findings preliminarily supported the hypothesized relationships.

### 4.1. Testing Hypotheses 1 and 2

Table 3 summarizes the multiple regression results. Hypothesis 1 posits that the relationship between overtime and subjective social status is negatively linear. The parameter estimates summarized in Table 3 showed that, as hypothesized, *β1* in Model 3 is statistically significant and negative (*β1* = −0.193, *p* < 0.05). Additionally, Oi explained 1.1% additional variance over and above that accounted for by the variables included in previous steps −ΔF = 4759, *p* < 0.05, supporting the salience of the negative relationship. Thus, H1 was supported. Hypothesis 2 posits that the relationship between overtime and social inclusion is a negatively linear. The parameter estimates summarized in Table 3 showed that, as hypothesized, *β1* in Model 4 is statistically significant and negative (*β1* = −0.235, *p* < 0.01). Additionally, Oi explained 2.1% additional variance over and above that accounted for by the variables included in previous steps −ΔF = 9.379, *p* < 0.01, supporting the salience of the negative relationship. Thus, H2 was supported.

### 4.2. Testing Hypotheses 3, 4, 5, and 6

**Figure 2:**
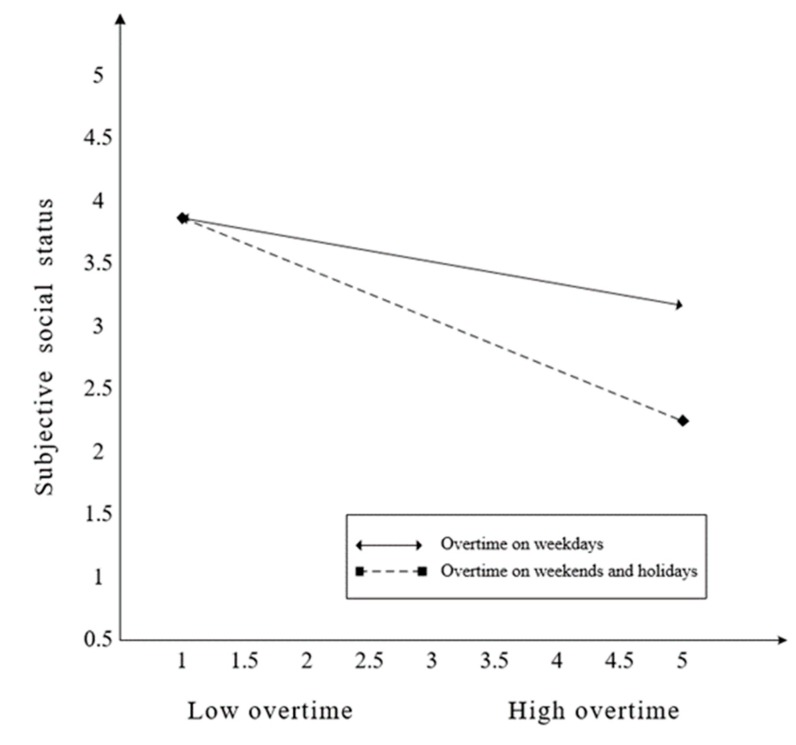
The moderating effect of time type between overtime and subjective social status.

**Figure 3:**
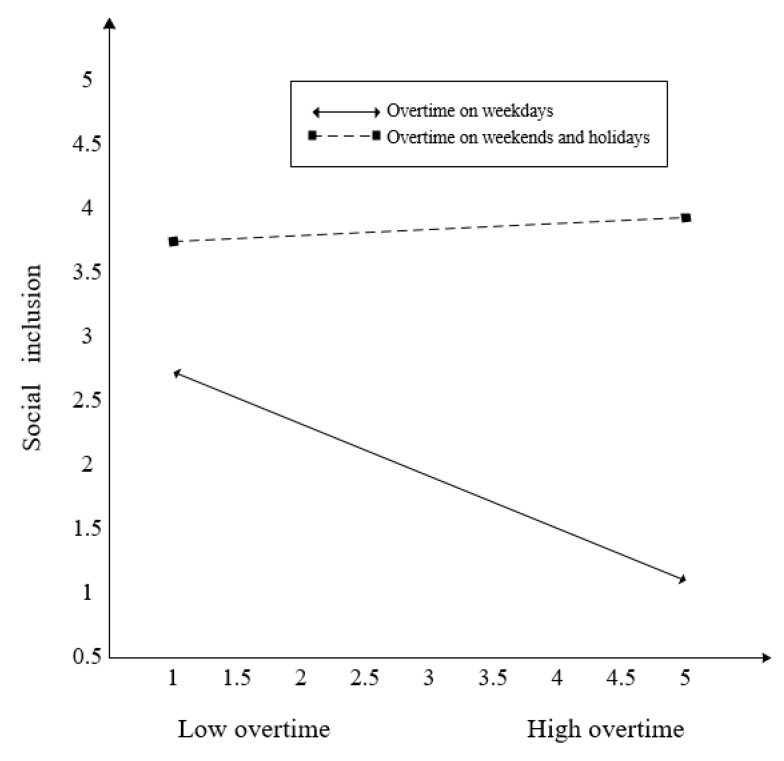
The moderating effect of time type between overtime and social inclusion.

Hypothesis 3 posited the moderating effect of time type on the relationship between overtime and subjective social status. The interaction term of overtime hours*time type on subjective social status was not significant (*β* = −0.228). The 95% confidence intervals indicate that zero was included ([−0.653, 0.232]). Thus, Hypothesis 3 was not supported. Besides, the effect of overtime on weekdays on subjective social status was not significant, −0.180 (Confidence Interval (CI) 95% = [−0.357, 0.006]), and the effect of overtime on weekends and holidays on subjective social status was not significant, -0.408 (CI 95% = [−0.786, 0.031]). Hypothesis 4 posited the moderating effect of time type on the relationship between overtime and social inclusion. The interaction effect of overtime*type on social inclusion was significant (*β* = 0.429). The 95% confidence intervals indicate that zero was not included ([0.065, 0.691]). Thus, Hypothesis 4 was supported. In addition, the effect of work overtime on weekdays on social inclusion was significant, −401 (CI 95% = [−0.565, −0.230]), and the effect of overtime on weekends and holidays on social inclusion was not significant, 0.028 (CI 95% = [−0.316, 0.207]). We plotted those interactions in Figure 2 and Figure 3. Although the moderating effect of time type was not significant, as shown in Figure 2, overtime predicted a lower subjective social status when employees work overtime on weekends and holidays rather than on weekdays. As shown in Figure 3, overtime significantly predicted lower social inclusion when employees work overtime on weekdays rather than on weekends and holidays.

**Figure 4:**
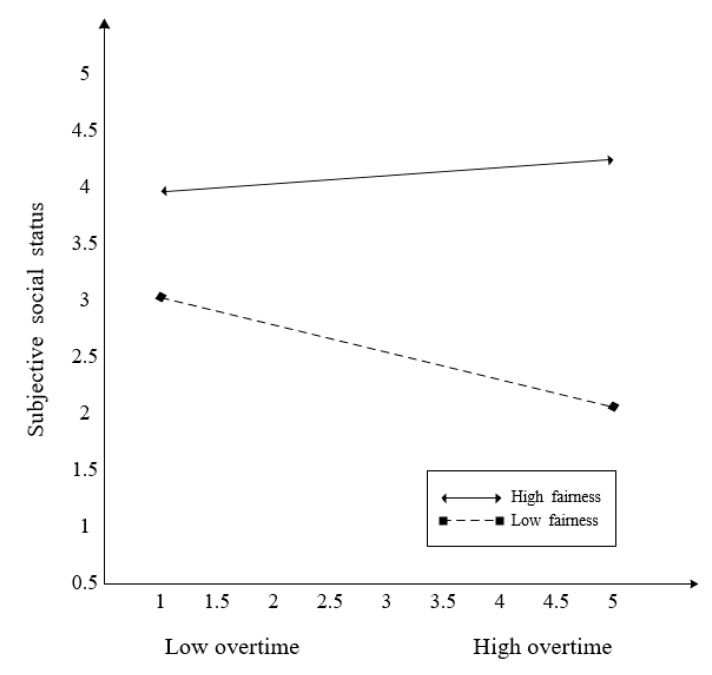
The moderating effect of fairness between overtime and subjective social status.

**Figure 5:**
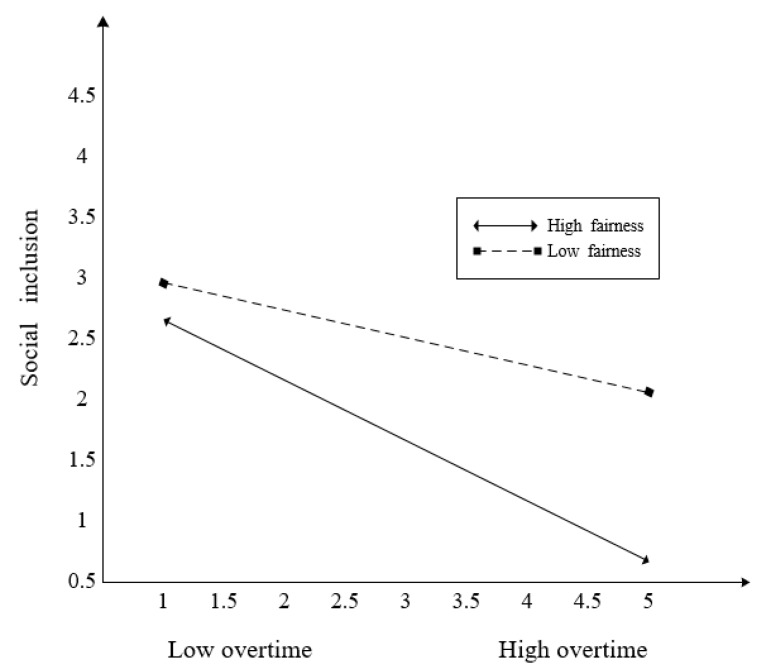
The moderating effect of fairness between overtime and social inclusion.

Hypothesis 5 posited the moderating effect of fairness on the relationship between overtime and subjective social status. The interaction effect of overtime * fairness on subjective social status was significant (*β* = 0.170). The 95% confidence intervals indicate that zero was not included ([0.039, 0.294]). Thus, Hypothesis 5 was supported. In addition, to interpret this interaction, we computed simple slopes between these variables at two levels of fairness (1 SD above the mean and 1 SD below the mean) [55]. As shown in Figure 4, overtime was significantly and negatively associated with subjective social status when fairness was low (*β* = −0.256, (CI 95% = [−0.405, −0.077]), but not when it was high (*β* = 0.064, (CI 95% = [−0.181, 0.283]). Hypothesis 6 posited the moderating effect of fairness on the relationship between overtime and social inclusion. The interaction effect of overtime * fairness on social inclusion was significant (*β* = −0.150), and the 95% confidence intervals indicated that zero was not included ([−0.266, −0.010]). However, the results showed that the negative relationship between working hours and social inclusion would be stronger at a high level of fairness rather than at a low level of fairness. Thus, Hypothesis 6 was not supported. As shown in Figure 5, overtime was significantly and negatively associated with social inclusion when fairness was high (*β* = −0.511, (CI 95% = [−0.722, −0.299]) rather than low (*β* = −0.228, (CI 95% = [−0.416, −0.064]).

## 5. Discussion

Previous research has focused on the significant adverse effects of long working hours on health-related outcomes such as mortality, circulatory disease, diabetes mellitus, metabolic syndrome, depressive state, anxiety, and sleep [8]. To deepen the research on working hours, in our study, we examined the negative effects of overtime working hours on victims’ social cognition, as well as the contingency factors which act on the relationships mentioned above. Based on the relative deprivation theory, we found that overtime working hours were negatively related to two types of crucial social perception—subjective social status and social inclusion. Specifically, our findings suggest that workers who have long hours are more likely to underestimate their social class perception and feel less social inclusion. Furthermore, our findings indicated that the relationship between overtime working hours and subjective social status is more influential among workers who think society is unfair, which suggests that the sense of unfairness may act as a catalyst that makes employees more sensitive to the relative deprivation caused by overtime. Finally, we found that the type of overtime also moderated the relationship between overtime working hours and social inclusion.

### 5.1. Theoretical Implications

This study makes several significant contributions. First, our findings indicate that work overtime can intensify the sense of relative exploitation and thus attenuate a subject’s perception of social status and social inclusion. In view of the increasing popularity of long working hours in contemporary organizations, people mainly discuss the health hazards of this work. However, the findings of this study broaden researchers’ understanding of working overtime by exploring its effect on social-related outcomes. Previous studies paid more attention to workers’ health from the view of “individual centric,” while our research focuses on their social cognitions from the view of “collect centric.” Moreover, to the best of our knowledge, this study is the first to explore the influences of working overtime on workers’ social consequences such as social status and social inclusion.

Second, our study provides insights into the idea of “worse off” described by the relative deprivation (RD) theory [24]. In particular, we found that the relationship between overtime working hours and social inclusion was stronger when working overtime on weekdays. According to Maslow’s arguments, the human basic needs can be classified in a hierarchy, and motivation links these needs with general behaviors [56]. The hierarchical structure of demand is physiological demand, security demand, belonging demand, respect demand, and self-actualization demand. We can also distinguish the first three kinds of demand as insufficient demand and the other needs as growing demand. For overtime, effort-recovery theory [57] suggests that overtime work can lead to a situation of prolonged insufficient recovery that is assumed to disturb physiological processes and, as a consequence, induce health problems [58]. The rest time on weekdays can meet the needs of workers’ recovery, which meets their deficiency needs. In contrast, both mass media and research have shown that leisure lifestyle is the common feature of the rich and famous, which meets their growth needs. The lack of deficiency needs would dominate individuals’ motivation and social cognition. Our findings support this inference. In the case where recovery time is not satisfied when working overtime on weekdays, the negative relationship between overtime hours and social inclusion will be strengthened.

Third, this study has advanced our understanding of relative deprivation theory by integrating the job demands and effort-reward fairness (e.g., [59,60]). Relative deprivation theory states that inequality manifests through various forms of socioeconomic comparisons (i.e., income inequality). However, the benefit of fair treatment in reducing the negative consequences of work overtime has received less attention (especially regarding fairness as a moderator of the effect of working overtime on workers), even though it is potentially more valuable [53,61]. We examined effort-reward fairness as a moderator of the effect of overtime working hours on subjective social status, which further broadens our understanding of the potential benefits of fair treatment in working overtime. Specifically, we found that effort-reward fairness moderated the negative relationship between overtime work and subjective social status, and the relationship was more salient when fairness perceptions are low rather than high. In addition, the results of this study enrich the overtime work literature. 

Fourth, we examined the moderating role of effort–reward fairness on the effect of overtime work on social inclusion but found that fairness failed to mitigate the negative effect of working overtime. Contrary to our hypothesis, our results appear to be inconsistent with previous research of fairness by showing that the negative association between overtime working hours and subjective social status was strengthened under the sense of high fairness. The existing literature suggests that in the process of employer–employee exchange, employees who feelthey are treated fairly are more likely to perform better in responding to job requirements than those who feel under-rewarded [59]. We suspect that the results of this study may be due to fairness sensitivity. In other words, when employees are always in an unfair environment, their sensitivity to fairness may be reduced, and thus the negative impact of overtime on their perception of social inclusion will be alleviated. Schulte-Braucks and his colleagues proposed that the effects of unreasonable and unnecessary tasks on employees’ self-esteem and counterproductive work behaviors are stronger among employees who are highly sensitive to injustice [62]. Judicial sensitivity concerns people’s response to unfair treatment [63]. We suggest that employees who are rarely mistreated may be more sensitive to fairness issues. Those who are highly sensitive to justice may explain unreasonable work arrangements from the perspective of justice, evoke a sense of relative deprivation, be angry at injustice, and underestimate their access to resources and institutions. On the contrary, people who are used to being abused are less likely to think the situation is unfair. For them, potential unfair cases are less emotionally disturbing or cognitively absorbing, and they are less motivated to act against them.

### 5.2. Practical Implications

This study provides evidence for institutions, employers, and employees about the negative influences of work overtime on the social perception of workers. The administrative department should recognize the importance of maintaining the social attitude of workers because the productivity of workers is the support of social and economic development and improvement. The labor bureau should strive to establish standard working hours as a favorable step to promote good social psychology and well-being of workers. 

Furthermore, both employees and employers should also be aware of the dangers of working long hours. For employees, working long hours is bad for their work–family balance, harms their health and well-being, and raises a sense of social alienation. For employers, they should realize that working long hours, as an unreasonable work arrangement, is not necessarily effective, which damages the long-term performance of the enterprise. In terms of work schedule, the managers should reasonably arrange the working time to protect employee’s rest and leisure time, especially on the weekdays. In terms of social responsibility, companies should ensure that employees have time and energy to spend with their families and fulfill their family roles. In doing so, employees can not only get a sense of responsibility but also obtain support from the family. In terms of regulations, enterprises should establish a fair workplace climate and enhance the psychological ownership of employees. In terms of training, it is necessary to cultivate the self-regulation ability of employees and help them deal with the unfavorable situation in a positive way.

### 5.3. Limitations

Although this study has a number of strengths, the limitations of this study should be acknowledged. First, given the data from the China Labor-Force Dynamics Survey (CLD), we can only use explicit variables rather than latent variables in our study. We acknowledge that the shortened items may not be as reflective of their theoretical concepts as the original scales are, which inflated their measurement error for parameter estimation. However, this single item approach has been used and examined in previous studies in China that have reported strong reliability and validity [64]. We believe that the use of secondary data has not resulted in any measurement concern that would invalidate our findings. Nevertheless, future studies may find it fruitful to utilize full scales to measure all of the variables tested in this study. Second, because of the cross-section data collected by CLD, we cannot apply a longitudinal research design, so the current design does not allow conclusions about absolute causality. A longitudinal design is needed to address the causal direction of the relationships among the variables. Third, the relationships among variables measured from the same source might have been inflated by common method variance (CMV) [65]. Beyond addressing these limitations, future research could also expand the present model by considering other moderators and mechanisms to enrich our model.

## 6. Conclusions

Employees who often work overtime or with a high workload are the types of workers that most organizations desire. Our findings suggest that overtime work may also damage workers’ social perception. Moreover, we also found that employees who work overtime on weekdays are less likely to integrate into society. Employees with a high sense of fairness are more likely to regard busy and overworked lifestyle as an aspirational social status symbol. Working overtime is not only a problem faced by enterprises but also a topic of general concern in society. We expect that our findings can highlight the critical role of overtime work in social life and also provide novel insights into social intervention aimed at the happiness and harmony of society.

## Figures and Tables

**Table 1:** Definition and description of variables in the study.

Variable Name	Description
**Control Variables**	
Gender	1 = Male; 2 = Female
Age	Years
Marriage	Your marital status is: 1 = Unmarried; 2 = First marriage; 3 = Remarried; 4 = Divorced; 5 = Bereaved; 6 = Cohabitation
Annual income	Your total income in 2015 was (including agricultural income, wage income, operating income, etc.) RMB (Yuan).
Living type	Your living type is 1 = Rural, 2 = Urban
Overtime location	Usually, where do you work overtime? 1 = Workplace; 2 = Other places outside the workplace (such as dormitory, home, hotel, etc.)
**Independent Variable**	
Overtime	How many hours did you work overtime last month?
**Dependent variables**	
Subjective social status	In our society, some people are at the top, and some are at the bottom. There is a ladder from top to bottom on the card below. The highest “10” is the top, and the lowest “1” is the bottom. At which level do you think you are?
Social inclusion	Are you likely to settle here in the future? 1 = Very unlikely; 2 = Less likely; 3 = Not sure; 4 = More likely; 5 = Very likely
**Moderating variables**	
Overtime type	In general, which of the following situations do your overtime work meet? 0 = Overtime on working days; 1 = Overtime on rest days or legal holidays
Fairness	Do you think your current standard of living is fair with respect to your efforts at work? 1 = Completely fair; 2 = Fairly; 3 = Not fair but not unfair; 4 = Relatively unfair; 5 = Totally unfair

**Table 2:** Standard Deviations and Correlations Among Study Variables.

Variable	Mean	SD	1	2	3	4	5	6	7	8	9	10
1. Age	35.62	9.495										
2. Gender	1.39	0.488	−0.100 *									
3. Marriage	1.88	0.804	0.338 **	−0.028								
4. Income	57,389.37	58,828.040	0.001	−0.204 **	0.119 *							
5.Unit type	1.77	0.423	−0.029	0.013	−0.001	0.177 **						
6. Overtime type	1.30	0.465	−0.140 **	0.011	0.010	−0.057	0.053					
7. Time type	0.20	0.397	0.036	−0.042	0.011	0.028	−0.166 **	0.019				
8. Hours of overtime	40.43	39.905	−0.019	−0.057	−0.023	−0.097	−0.196 **	−0.037	−0.064			
9. Fairness	3.17	0.944	−0.100 *	0.011	0.017	0.074	−0.065	−0.264**	−0.047	−0.130 **		
10. Social inclusion	3.23	1.585	0.003	−0.041	0.032	−0.211 **	−0.248*	0.119*	0.156**	−0.204 **	0.004	
11. Subjective social status	4.11	1.784	0.009	−0.007	0.072	0.249 **	0.055	−0.053	0.057	−0.132 **	0.200 **	0.200 **

Note: SD= standard deviation; N = 400; *p < 0.05. **p < 0.01.

**Table 3:** Unstandardized regression coefficients.

Variables	Subjective Social Status Model 1	Social Inclusion Model 2	Subjective Social Status Model 3	Social Inclusion Model 4
Constant	3.816 **	1.292 **	4.020 **	1.541 **
Age	0.009	−0.021	0.004	−0.027
Gender	0.166	−0.028	0.136	−0.063
Marriage	0.095	0.027	0.093	0.025
Income	0.443 **	0.281 **	0.427 **	0.262 **
Unit type	0.053	0.788 **	−0.029	0.688 **
Overtime type	−0.157	0.410 *	−0.169	0.395*
Hours of overtime			−0.193 *	−0.235 **
R	0.260	0.324	0.280	0.354
R2	0.067	0.105	0.079	0.126
ΔR2		0.105	0.011	0.021
F	4.734 **	7.662 **	4.776 **	8.048 **
ΔF			4.759 *	9.379 **

Note: *p < 0.05. **p < 0.01.
